# Supplementary material for: Analysis of interaction risks of patients with polypharmacy and the pharmacist interventions performed to solve them—A multicenter descriptive study according to medication reviews in Hungarian community pharmacies
Source: PLoS One. 2021 Jun 22;16(6):e0253645. doi: 10.1371/journal.pone.0253645 (PMC8219127; doi:10.1371/journal.pone.0253645)
Supplement: Attachment — Submitted filename: PON-D-21-08091.docx [file pone.0253645.s001.docx]

The study on drug-related problems, especially the interaction risks of patients with polypharmacy entering community pharmacies in terms of their nature, and clinical severity and the pharmacist interventions performed to solve them. It appears to be a good study because of the increasing number of chronic conditions and the need to use a number of medications, which increases the risk of drug-related problems and reduces medication adherence, further halting the overall outcome. The authors of this study correctly identified the problems and devised solutions to address them. Because HCPs and patients may lack knowledge or awareness about DRPs, they provide education before beginning the project.

However, there are issues need to be addressed before it can be considered for publication.

**Abstract**

Kindly make structure abstract, objective, methodology, results and conclusion

**Introduction**

It will be good to talk about the need of the study? What is the rational for the study? From previous literature available from Hungry or other countries, if available such data. Please highlighted in the introduction part.

**Methods**

Excellent methodology and literature review however the authors miss about the researcher, how they trained or one of the author/researcher involve in data collection and analysis?

There is a need to describe the basic country geography, such as how many counties/cities/towns there are, and so on.

Which methodology was used to select participants to represent the study across the country?

Is a sample size calculation performed prior to the actual study? What is the starting point for including 78 pharmacists, 98 general practitioners, and 755 patients?

Please explain in methodology section

**Ethics approval**

Why didn't the researchers apply to the Human Research Ethics Committee (HREC)?

All research projects involving human participants, including the use of non-face-to-face data collection methods such as online surveys and postal surveys, require HREC approval.

I understand that ethical approval is not required in their country, as researchers stated.

I believe they should still apply for HREC; the HREC committee may grant them exemption based on the project is negligible risk.

**General comments**

kindly check for typographical error

Please follow uniform pattern for reference style, refer journal guidelines

The strength and the limitations of the study? Make it as separate heading

I think if the corrections are made the paper can be accepted for publication in PloS ONE.
