# Supplementary material for: Analysis of interaction risks of patients with polypharmacy and the pharmacist interventions performed to solve them—A multicenter descriptive study according to medication reviews in Hungarian community pharmacies
Source: PLoS One. 2021 Jun 22;16(6):e0253645. doi: 10.1371/journal.pone.0253645 (PMC8219127; doi:10.1371/journal.pone.0253645)
Supplement: Attachment — Submitted filename: reviewer comments.docx [file pone.0253645.s002.docx]

1.	Please correct the spelling of “data” in data availability statement

2.	Abstract of the current study requires particular attention. Authors should concisely describe the methodology, number of participants, primary outcomes, major findings. Moreover, conclusion must be align with the findings with specific remarks

3.	This manuscript needs considerable efforts to improve the scientific writing. Though authors provided the sentences with good English, but still essence of scientific writing is missing in the draft.

4.	I will suggest authors to make heading of “Operational definitions” and put all the terminologies under this heading

5.	The methodology section requires appropriate demarcation i.e. study design, sample size estimation, study duration, study population with inclusion and exclusion criterion, data collection, study variables. There is no detail of statistical analysis in the draft.

6.	Please provide the English translation of reference 44; I will suggest authors to explain bit about the DRPs classification as provided reference is not in English.

7.	Please provide the operational definition of polypharmacy used in the current study

8.	The presentation of figure 3 is confusing, can authors use another format to present the result e.g. stacked column or stacked bar

9.	Can authors provide relationship analysis between demographic variables with severity of DRPs. I am convinced that classification of DRPs based on severity or necessity for intervention is of great value. Pharmacists can ascertain the particular class of patients vulnerable to such DRPs and could avoid such instances in the future.

10.	In 755, 984 DRPs were reported, indicating that all the patients visiting the pharmacies experienced DRPs. It is not clear that authors took data of all patients admitting to pharmacies during the specified time period or they took only those patients who had DRPs.

11.	During the 6 months duration of the study, only 755 patients attended the pharmacies located in 35 settlements?

12.	15 out of 20 countries???? This statement is not clear

13.	Please provide the details of covered area so generalizability of the findings can be assessed.

14.	Were all these pharmacies included in the current study belong to same owner or cooperation so they were randomly selected?

15.	The pharmacy selection criteria are needed to be explained as authors claimed that current study has nationwide coverage.

16.	How the sampling was done in the current study? Who collected the data? How DRPs identified (by pharmacist or by software)?

17.	Authors need to discuss about the possible bias in the current study

18.	Conclusion of the study needs attention, it should be specific to the findings and with future suggestions

19.	6. The incidence of pharmacist interventions (Table 4) to solve interaction risks, in order of …. There is no need to indicate table number in the figure legend

20.	Since warning of GP and education was most common intervention in the current study, authors need to underscore the reason behind that as very less data is available showing the role of pharmacist as dosage modifier or drug changer? Do pharmacists consider therapy modification bit risky and challenging as compared to other interventions?
